# Identification of heart rate dynamics during treadmill exercise: comparison of first- and second-order models

**DOI:** 10.1186/s12938-021-00875-7

**Published:** 2021-04-21

**Authors:** Hanjie Wang, Kenneth J. Hunt

**Affiliations:** Department of Engineering and Information Technology, Division of Mechanical Engineering, Institute for Rehabilitation and Performance Technology, Bern University of Applied Sciences, 3400 Burgdorf, Switzerland

**Keywords:** Heart rate dynamics, System identification, Treadmills

## Abstract

**Background:**

Characterisation of heart rate (HR) dynamics and their dependence on exercise intensity provides a basis for feedback design of automatic HR control systems. This work aimed to investigate whether the second-order models with separate Phase I and Phase II components of HR response can achieve better fitting performance compared to the first-order models that do not delineate the two phases.

**Methods:**

Eleven participants each performed two open-loop identification tests while running at moderate-to-vigorous intensity on a treadmill. Treadmill speed was changed as a pseudo-random binary sequence (PRBS) to excite both the Phase I and Phase II components. A counterbalanced cross-validation approach was implemented for model parameter estimation and validation.

**Results:**

Comparison of validation outcomes for 22 pairs of first- and second-order models showed that root-mean-square error (RMSE) was significantly lower and fit (normalised RMSE) significantly higher for the second-order models: RMSE was 2.07 bpm ± 0.36 bpm vs. 2.27 bpm ± 0.36 bpm (bpm = beats per min), second order vs. first order, with $p = 2.8 \times 10^{-10}$; fit was $54.5\% \pm 5.2$% vs. $50.2\% \pm 4.8$%, $p = 6.8 \times 10^{-10}$.

**Conclusion:**

Second-order models give significantly better goodness-of-fit than first-order models, likely due to the inclusion of both Phase I and Phase II components of heart rate response. Future work should investigate alternative parameterisations of the PRBS excitation, and whether feedback controllers calculated using second-order models give better performance than those based on first-order models.

## Background

Characterisation of heart rate (HR) dynamics with respect to changes in exercise intensity provides models that can be used to synthesise control algorithms to maintain target HR levels [1]. The control of HR is important in the design of training protocols that aim both to maintain and to improve cardiorespiratory fitness; this applies to healthy individuals [2] and also in different patient populations [3, 4]. Target heart rate profiles come in various forms such as high-intensity interval training (HIIT) that repeats high-intensity exercise connected by low-intensity recovery intervals; HIIT has potential to enhance cardiovascular health and fitness when compared to training at constant work rates (systematic reviews: [5, 6]).

Recent work investigated the effect of exercise intensity and time on HR dynamics using first-order models [7], but it may be beneficial to include higher order effects: based on physiological study, the dynamics of both oxygen uptake and HR responses to changes in exercise intensity are known to have three distinct phases [8]. These are: (i) a Phase I component lasting $\sim$ 15 s with a relatively small-magnitude ventilatory response, but where HR can increase by about 50% of its total response [9]; (ii) a Phase II component between around 15 s and 3 min contributing the further increase of cardiopulmonary response; and then, (iii) if the applied exercise intensity exceeds the anaerobic threshold, a Phase III component is prolonged and rises slowly. The three components can each be modelled as single exponentials (first-order systems) each with their own time delay, gain, and time constant [10]. In addition to these primary dynamic responses, the phenomenon of heart rate variability (HRV) can be added to the model to represent the regulatory activities of the autonomic nervous system; in the context of feedback control of HR, HRV represents a broad-spectrum disturbance term [1].

Because it can be challenging to estimate the separate Phase I and II components using data which is noisy, those two phases have often been identified as a combined single exponential model with a time constant termed the mean response time (MRT, [8]), which is effectively the first-order approach taken in the previous studies that focused on system identification [7] and feedback control [1]. In feedback control, the slow Phase III component can readily be neglected as it is compensated by inclusion of an integrator in the controller. The focus of the present work is therefore the investigation of whether the separate identification of Phase I and II components, i.e., the employment of a second-order model, can give better model fidelity.

Other recent approaches to HR dynamics identification focused mainly on the modelling of the Phase II and III components of the HR response. Several studies employed a non-linear state-space model structure comprising two different states ($x_1$ and $x_2$) to separately describe the Phase II and Phase III dynamics [11–15]. Other work used linear time-varying systems to model the slow Phase III dynamic [16, 17]. While inclusion of Phase III may improve overall model fidelity, it will, as noted above, have negligible impact on feedback-control performance as it will be eliminated by the integral action. In contrast, it can be anticipated that separate modelling of the Phase I and II components might lead to better control performance when the model is used as the basis of an analytical, model-based feedback design.

To this end, this work aimed to investigate whether second-order models with separate Phase I and Phase II components of HR response can achieve better fitting performance compared to first-order models that do not delineate the two phases. Here, an input signal of PRBS (pseudo-random binary sequence) form was designed to excite both the Phase I and Phase II components.

## Results

**Fig. 1:**
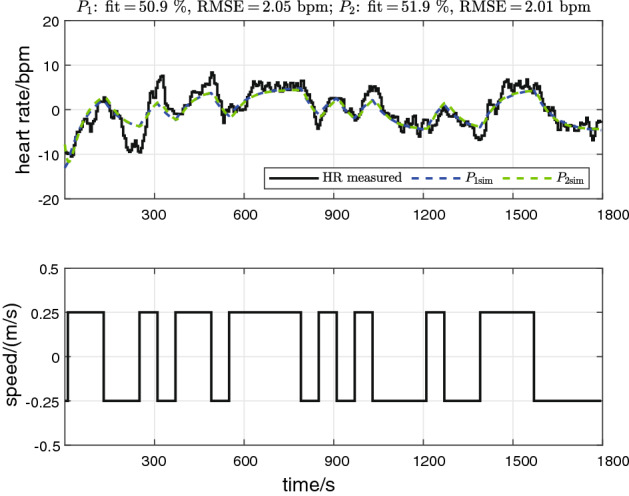
Data preprocessing and model validation: exemplary data for participant P04 (the raw data for this test are shown in section ‘Method’). Upper plot: HR measurement from validation data set after detrending (solid black line), simulated HR response of first-order model ($P_{1 \mathrm{sim}}$, blue dashed line), and simulated HR response of second-order model ($P_{2 \mathrm{sim}}$, green dashed line). Lower plot: treadmill speed from validation data set after mean removal

To illustrate the procedures of data preprocessing and model validation, an exemplary result from participant P04 is shown (Fig. 1); the raw data for the same participant are shown above below in the section ‘Method’. For this example, the second-order model $P_2$ gave better performance than the first-order model $P_1$: fit was 51.9% vs. 50.9% ($P_2$ vs. $P_1$) and RMSE was 2.01 bpm vs. 2.05 bpm.

**Table 1:** Overall outcomes for first- and second-order models and comparison of outcome differences (see also Fig. 2)

	Mean ± SD		MD (95% CI)	*p*-value
	$P_1$	$P_2$	$P_2 - P_1$	
RMSE/bpm	2.27 ± 0.36	2.07 ± 0.36	$-0.19$ ($- \infty$, $-0.16$)	2.8 $\times$ $10^{-10}$
fit/%	50.2 ± 4.8	54.5 ± 5.2	4.3 (3.6, $+ \infty$)	6.8 $\times$ $10^{-10}$

$$n = 22$$

$P_1$ first-order models, $P_2$ second-order models, *SD* standard deviation, *MD* mean difference, *95% CI* confidence interval for the mean difference , *p-value* paired one-sided t tests, *RMSE* root-mean-square error, *fit* normalised root-mean-square error, *bpm* beats per min

**Fig. 2:**
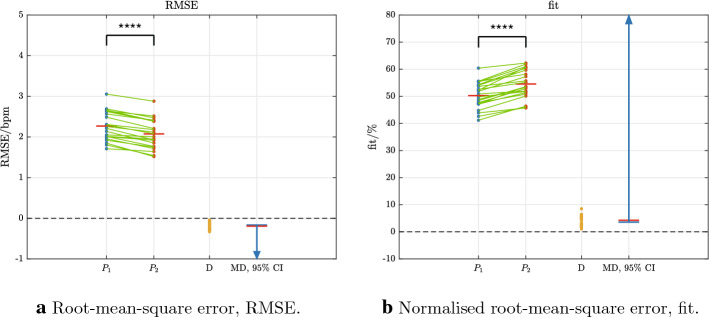
Primary outcomes: data samples and differences for RMSE and fit between 22 first-order models, $P_1$, and 22 second-order models, $P_2$ (see also Table 1). Sample pairs for each participant are connected by green lines; mean values are shown as red horizontal bars (with numerical values given in Table 1). Sample-pair differences are shown as D ($P_2 - P_1$). The mean difference (MD) is depicted as a red bar and the blue arrow is the corresponding 95% confidence interval (CI). For both RMSE and fit, the 95% CI does not contain the value 0, thus showing a significant improvement for $P_2$ vs. $P_1$ ($p < 0.05$, Table 1; the notation **** denotes $p < 0.0001$)

The overall statistical comparison of validation outcomes for the 22 pairs of first- and second-order models showed that RMSE was significantly lower and fit significantly higher for the second-order models: RMSE was 2.07 bpm ± 0.36 bpm vs. 2.27 bpm ± 0.36 bpm, $P_2$ vs. $P_1$, with $p = 2.8 \times 10^{-10}$ (Table 1; Fig. 2a); fit was $54.5\% \pm 5.2$% vs. $50.2\% \pm 4.8$%, $p = 6.8 \times 10^{-10}$ (Table 1; Fig. 2b). The graphical illustration of overall outcomes (Fig. 2) shows how widely individual samples and their differences are dispersed, together with means and their 95% confidence intervals (CIs). These plots allow visual determination of significant differences, if they exist: whenever there is a significant difference, the value 0 will not be contained within the corresponding CI.

The sample size was estimated a priori by a statistical power calculation that used estimates of expected effect sizes and sample standard deviations, with significance level set to 5% ($\alpha$ = 0.05) and with a statistical power of 80% ($1-\beta$ = 0.8).

The observed outcomes show large effect sizes (approximately 9% for both outcomes) and extremely low *p* values (on the order of $10^{-10}$), thus pointing to a well-powered statistical analysis. In fact, post hoc statistical power analysis based on observed effect sizes and sample dispersions gives an observed power of 100% for both outcomes.

**Fig. 3:**
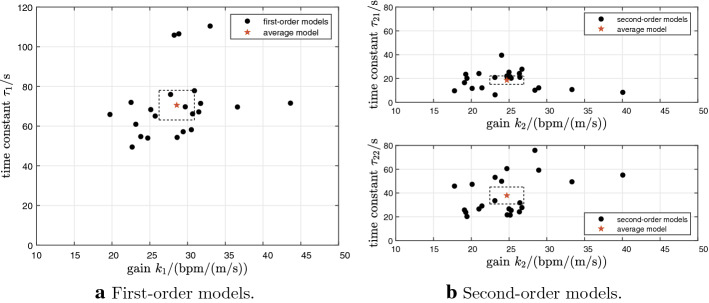
Dispersion of estimated model parameters for 22 first- and 22 second-order models. The stars depict the average models. The 95% confidence intervals for the mean gains and time constants are shown as rectangular boxes

A graphical illustration of the dispersion of estimated model parameters for the 22 first- and 22 second-order models is provided (Fig. 3). The overall first- and second-order models were obtained by averaging the individual gains and time constants. For the first-order models, the overall gain was $k_1$ = 28.57 bpm/(m/s) ± 5.27 bpm/(m/s) (mean ± standard deviation) while the time constant was $\tau _1$ = 70.56 s ± 16.84 s. For the second-order models, the overall gain was $k_2$ = 24.70 bpm/(m/s) ± 5.07 bpm/(m/s) and the overall time constants were $\tau _{21}$ = 18.60 s ± 7.88 s and $\tau _{22}$ = 37.95 s ± 16.01 s. This gives the average transfer functions for first- and second-order models as follows:

1 $$\begin{aligned} u \mapsto y \negthickspace : \; P_1(s)= & {} \frac{28.57}{70.56 s + 1}, \end{aligned}$$

2 $$\begin{aligned} u \mapsto y \negthickspace : \; P_2(s)= & {} \frac{24.70}{(18.60 s + 1)(37.95 s + 1)}. \end{aligned}$$

## Discussion

This study aimed to investigate whether second-order models with separate Phase I and Phase II components of heart rate response can achieve better fitting performance compared to first-order models that do not delineate the two components. The results clearly demonstrate that second-order models give significantly better goodness-of-fit, in terms of both RMSE and fit (NRMSE): RMSE was on average 0.19 bpm lower and fit 4.3% higher for the second-order model structure (*p* values were on the order of $10^{-10}$ in both cases); that these significance levels were achieved with a small sample size of only 11 participants underline the difference.

The approach taken here focused on control-orientated model structures, in the sense that the estimated models would be intended to be used for analytical (model-based) design of heart rate control systems. For this reason, slow Phase III components in the data were eliminated by detrending prior to parameter estimation. This is consistent with feedback-control scenarios where slowly drifting Phase III variations in heart rate are automatically compensated using integral action in the controller.

A further difference between the methodology employed here and heart rate modelling approaches taken in the physiological literature, [8], is that a nominal operating point was assumed, and small deviations around this point were considered (in this case, the operating point was set at the transition between exercise levels considered to be moderate and vigorous). This is consistent with linear feedback design approaches, which are implicitly based on models that are small-signal linearisations around an operating point; the purpose of feedback control is indeed to maintain the controlled variable, viz., heart rate, close to a target level.

For these reasons, it is not possible to compare the overall estimated model parameters (gains and time constants, Eqs. (1) and (2) with values given in the physiological literature (e.g., [9, 10]), because, there, responses are usually recorded using large steps from a resting or low-intensity baseline.

A consequence of the control-orientated methodology followed here is that the design of the PRBS input signal becomes important. For non-linear systems, it is known that the parameters of linear approximations are input dependent [18], which motivates further work to explore the effect of PRBS amplitude and frequency content on model fidelity; in particular, it is important to focus the information content on frequencies around the intended crossover band of the closed-loop system [19].

Future work should investigate whether the observed improvement in model fidelity translates into better feedback-control performance, i.e., whether controllers designed on the basis of second-order models perform better, in some sense, than those designed using first-order models. Because of the fundamental property of feedback that plant uncertainty (including modelling error) is reduced, the answer to this question will likely not be as clear cut as in the open-loop identification case.

## Conclusions

Second-order models give significantly better goodness-of-fit than first-order models, likely due to the inclusion of both Phase I and Phase II components of heart rate response. Future work should investigate alternative parameterisations of the PRBS excitation, and whether feedback controllers calculated using second-order models give better performance than those based on first-order models.

## Methods

### Participants

Eleven healthy participants were recruited (8 males, 3 females) with age 32.5 years ± 12.3 years (mean ± standard deviation), body mass 75.5 kg ± 14.4 kg, and height 179 cm ± 12 cm. For inclusion, each participant was required to be a regular exerciser (30-min bouts, 3 times per week) and non-smoker, and to be free of injury and illness.

### Test protocols

**Fig. 4:**
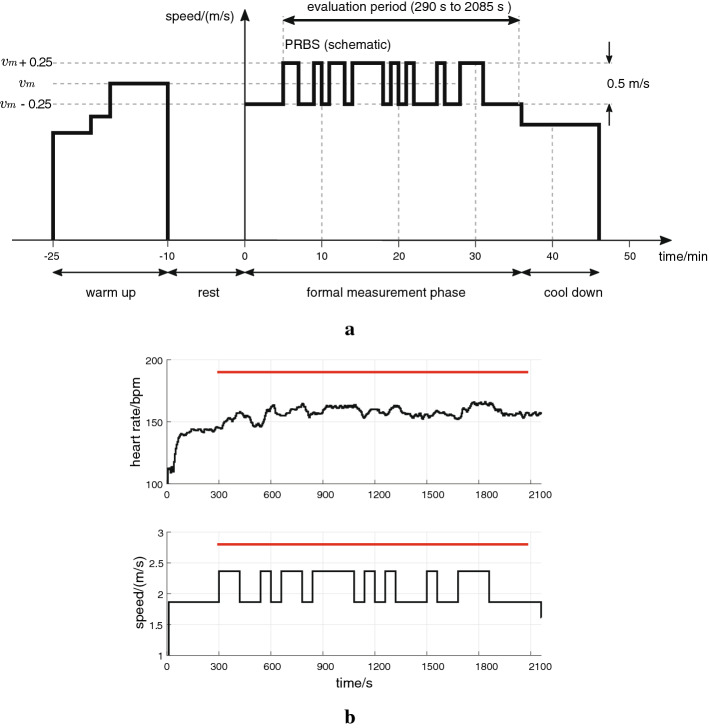
Identification test protocol. **a** Test phases and treadmill speed. **b** Original data record from one participant (P04; upper plot—HR measurement; lower plot—speed of the treadmill); the evaluation period is depicted by the red horizontal bar

To generate separate estimation and validation data sets, each participant took part in two identification tests; there was an interval of at least 48 h between the two tests. Before each test, participants were asked to meet the following requirements: refrain from strenuous activity for 24 h, caffeine for 12 h, avoid large meals for 3 h. Each test session had four phases: a 15 min warm up, a 10 min rest, a 36 min formal measurement, and a 10 min cool down (Fig. 4a).

In the warm up, a feedback-control system was employed to automatically regulate the speed of the treadmill to maintain a constant target HR. The target HR, denoted $\mathrm{HR}_{\mathrm{ref}}$, was computed individually for each participant and corresponded to the HR at the transition between intensity levels considered to be moderate or vigorous [3], as follows: $\mathrm{HR}_{\mathrm{ref}} = 0.765\times (220 - \mathrm{age})$ [beats/min, bpm] (except for participant P03, for whom the factor 0.7 was used, because 0.765 led to HR remaining in the vigorous-intensity regime). The mean speed of the treadmill during the final 2 min of the warm up phase was subsequently used as the mid-level speed, denoted $v_\text{m}$, for the next phase.

In the formal measurement phase, the speed of the treadmill, denoted *v*, was designed as a fifth-order PRBS with mean speed $v_{\rm m}$ and amplitude 0.25 m/s, i.e., $v = v_{\mathrm m} \pm 0.25~$ m/s (to illustrate, a single original data record is provided; Fig. 4b). Model parameter estimation and validation was performed over a full cycle of the PRBS using an evaluation period from 290 s to 2085 s (Fig. 4); the first 5 min were excluded to eliminate the initial transient. During the cool down phase, the speed of the treadmill was kept constant at $v = v_{\mathrm{m}} - 0.5~$ m/s.

### Equipment

All tests were carried out using a treadmill (model Venus, h/p/cosmos Sports & Medical GmbH, Germany) controlled by a PC running real-time Matlab/Simulink (The MathWorks, Inc., USA). HR recording was performed with a chest strap (H10, Polar Electro Oy, Finland) and a wireless receiver (Heart rate Monitor Interface, Sparkfun Electronics, USA) connected to the Simulink model via a serial port. HR measurements were received at a rate of 1 Hz and then downsampled to a sample rate of 0.2 Hz (sample period 5 s) by averaging consecutive batches of five individual samples.

### Data preprocessing, model identification, and outcome measures

As noted above, each participant completed two identification tests, thus generating individual data sets (I and II) for model parameter estimation and validation. To prevent over-fitting and to eliminate potential order-of-presentation effects, a counterbalanced cross-validation approach was implemented: for each participant, data set I was used to estimate model parameters and data set II was used as validation data for the estimated models; then, for the same participant, data set II was used for model estimation and data set I for validation. Thus, for the 11 participants, a total of 22 estimation data sets and 22 validation data sets were obtained.

According to the test protocol (Sect. 5.2, Fig. 4a), an evaluation interval from 290 s to 2085 s was used to estimate and validate model parameters. This interval, within one single PRBS period, was selected, such that the number of samples where the input was high ($v = v_{\mathrm{m}} + 0.25~$m/s) equalled the number of samples where the input was low ($v = v_{\mathrm{m}} - 0.25~$m/s). Here, on the evaluation period from 290 s to 2085 s and with a sample period of 5 s, the total number of samples was *N* = 360, thus giving 180 low samples and 180 high samples.

To remove any potential drifting Phase III dynamic of the HR response, the mean value and any trend were removed (Matlab “detrend” function) prior to estimation and validation; the mean value of the input signal was also removed. An exemplary data set following this preprocessing procedure is provided (Fig. 1), with raw data are shown above (Fig. 4b).

For each estimation data set, two linear time-invariant transfer functions were employed to model the dynamic response from treadmill speed to HR: a first-order transfer function (Eq. 3) which combined Phases I and II into a single time constant, and a second-order transfer function (Eq. 4) with separate time constants for Phases I and II. Hence, for the 11 participants, a total of 22 first-order models and 22 second-order models were estimated:

3 $$\begin{aligned} u \mapsto y \negthickspace : \; P_1(s)= & {} \frac{k_1}{\tau _1 s + 1}, \end{aligned}$$

4 $$\begin{aligned} u \mapsto y \negthickspace : \; P_2(s)= & {} \frac{k_2}{(\tau _{21} s + 1)(\tau _{22} s + 1)}. \end{aligned}$$

Here, $k_1$ and $k_2$ are steady-state gains, and $\tau _1$, $\tau _{21}$, and $\tau _{22}$ are time constants. Model parameters were calculated from the estimation data sets using a least-squares optimisation procedure (“procest” function from the Matlab System Identification Toolbox; The Mathworks, Inc., USA).

After model estimation, the corresponding validation data sets were used to compute goodness-of-fit measures for the resulting first- and second-order models. Two outcome measures were used: the normalised root-mean-square error [denoted fit, Eq. (5)], and the root-mean-square error [denoted RMSE, Eq. (6)], as follows:

5 $$\begin{aligned} \text {fit (NRMSE)} \; [\%]= & {} \left( 1-\sqrt{\frac{\sum _{i=1}^{N}( \mathrm{HR}(i)-\mathrm{HR}_{\mathrm{sim}}(i))^2}{\sum _{i=1}^{N}(\mathrm{HR}(i) - \overline{\mathrm{HR}})^2}} \right) \times 100~\%, \end{aligned}$$

6 $$\begin{aligned} \text {RMSE} \; [\mathrm{bpm}]= & {} \sqrt{\frac{1}{N}\sum _{i=1}^{N}(\mathrm{HR}_{\mathrm{sim}}(i) - \mathrm{HR}(i))^2}. \end{aligned}$$

Here, $\mathrm{HR}_{\mathrm{sim}}$ is the simulated HR response obtained using the estimated models and the input signal, and HR is the measured HR from the validation data. $\bar{\mathrm{HR}}$ is the mean value of ${\mathrm HR}$. *i* is the discrete time index and *N* is the number of discrete samples considered (as described above, $N = 360$). Both of the above outcomes were calculated using the “compare” function from the Matlab System Identification Toolbox.

### Statistics

Statistical analysis was performed to test the hypothesis that the goodness-of-fit outcomes of second-order models are better (higher fit and lower RMSE) compared to first-order models. Prior to analysis, normality of differences between the goodness-of-fit outcomes was formally assessed using the Matlab “lilliefors” function (this implements a Kolmogorov–Smirnov test with correction according to the Lillifors method). As it transpired that all differences were not significantly different from a normal distribution, paired one-sided t tests were employed for hypothesis testing. Hypothesis testing used a significance threshold of $5\%$ ($\alpha = 0.05$). The Matlab Statistics and Machine Learning Toolbox (The Mathworks, Inc., USA) was employed.

## Data Availability

The datasets used and/or analysed during the current study are available from the corresponding author on reasonable request.

## Abbreviations

bpm: Beats/min
CI: Confidence interval
fit/NRMSE: Normalised root-mean-square error
HR: Heart rate
HRV: Heart rate variability
MD: Mean difference
PRBS: Pseudo-random binary sequence
RMSE: Root-mean-square error
SD: Standard deviation
*k*: Steady-state gain
$$\tau $$: Time constant
